# Supplementary figures and images for: Sensor-Based Rehabilitation in Neurological Diseases: A Bibliometric Analysis of Research Trends
Source: Brain Sci. 2023 Apr 26;13(5):724. doi: 10.3390/brainsci13050724 (PMC10216556; doi:10.3390/brainsci13050724)

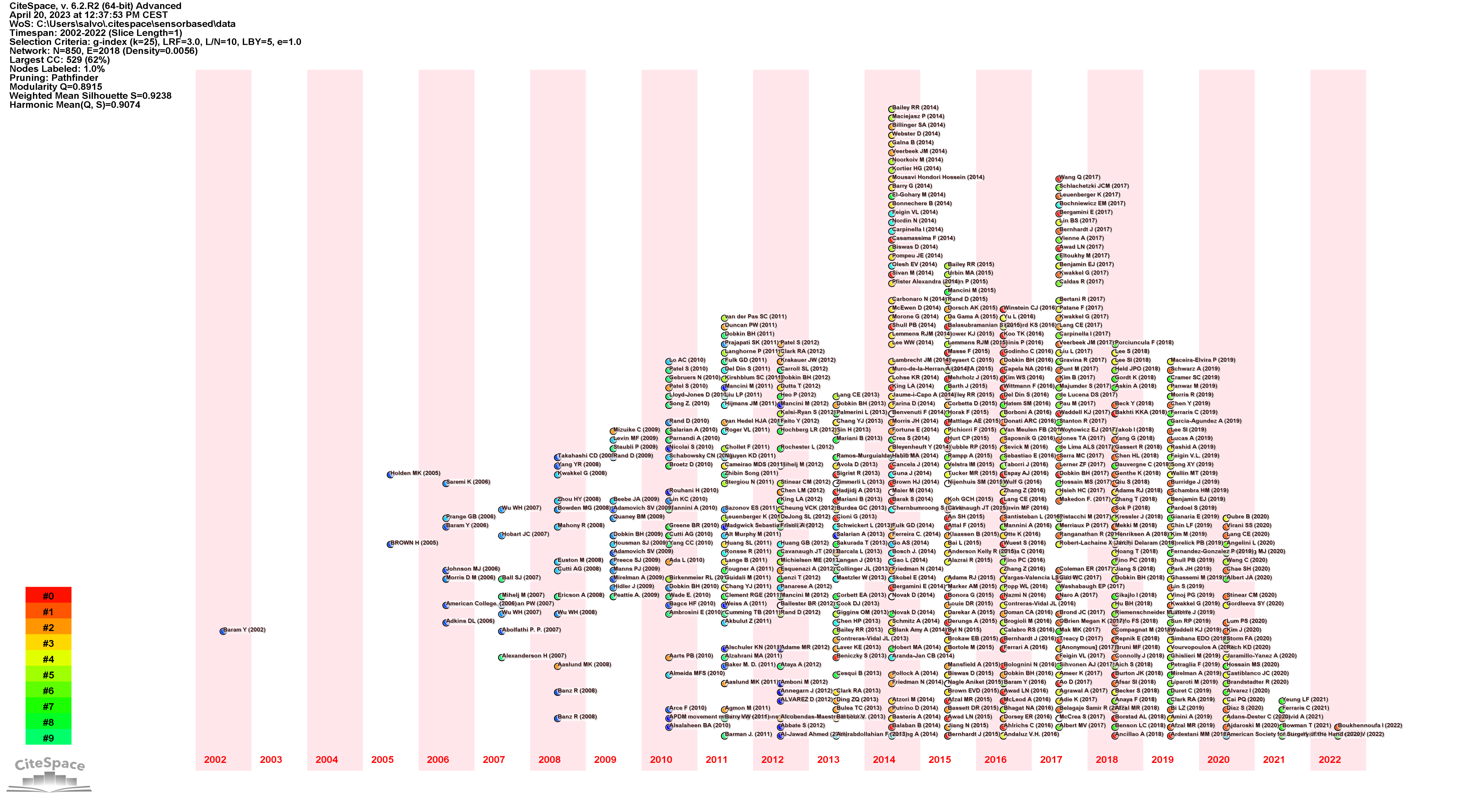

Supplement: Supplementary file 1 [file brainsci-13-00724-s001.zip › Figure S2.png]

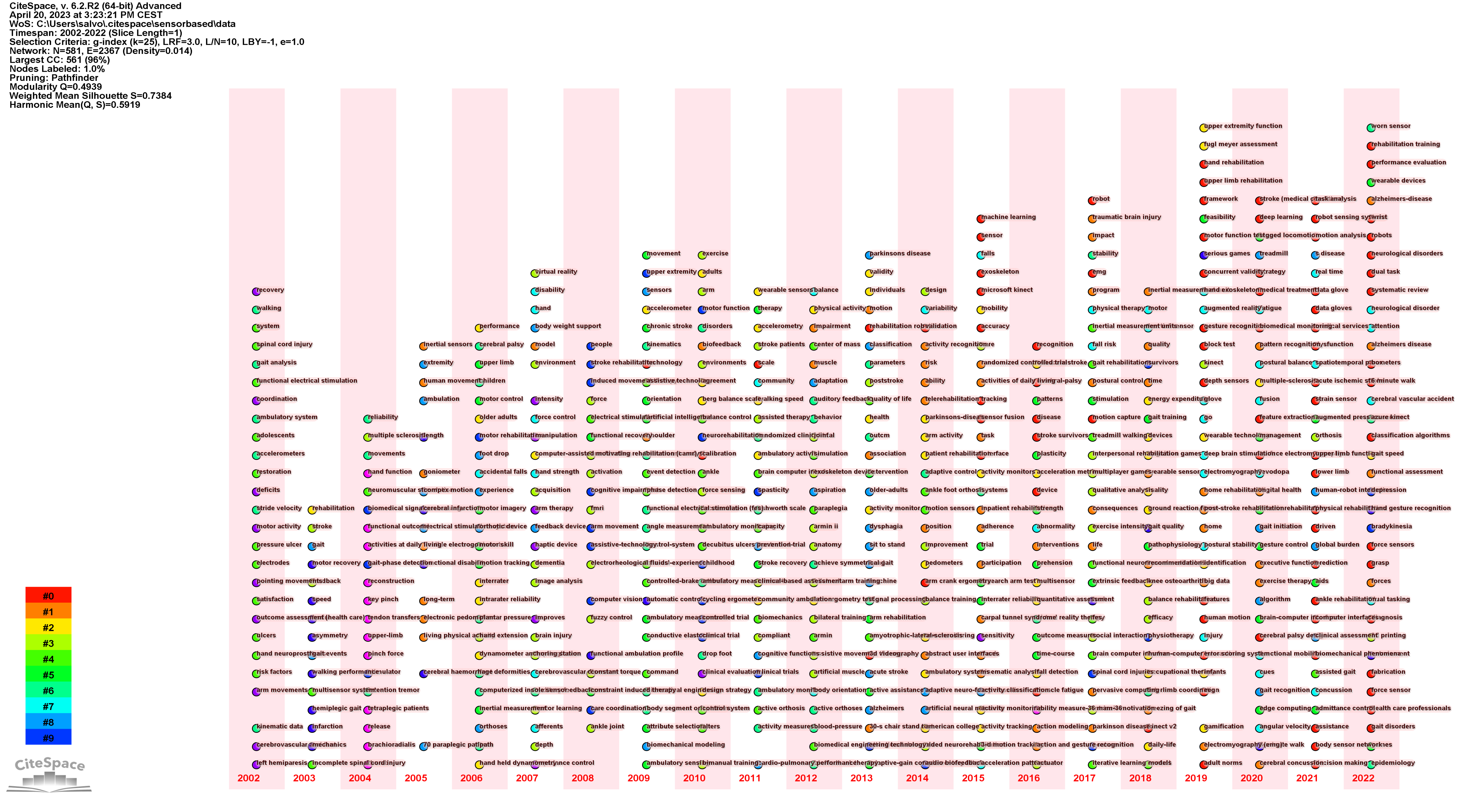

Supplement: Supplementary file 1 [file brainsci-13-00724-s001.zip › Figure S3.png]
